# Knowledge and practice of physicians during COVID-19 pandemic: a cross-sectional study in Lebanon

**DOI:** 10.1186/s12889-020-09585-6

**Published:** 2020-09-29

**Authors:** Linda Abou-Abbas, Zeina Nasser, Youssef Fares, Mohammad Chahrour, Rana El Haidari, Rola Atoui

**Affiliations:** 1 Neuroscience Research Center, Faculty of Medical Sciences Lebanese University, Hadat, Lebanon; 2 Endocrinologist, Al Zahraa Hospital University Medical Center, Beirut, Lebanon; 3 Environments and Health doctoral school, University of Bourgogne Franche-Comté, 25000 Besançon, France; 4 Infectious Disease Department, Lebanese University, Faculty of Medical Sciences, Hadath, Lebanon; 5 Infectious Disease Division Al Zahraa Hospital University Medical Center, Beirut, Lebanon

**Keywords:** COVID-19, Physicians, Knowledge, Practice, Lebanon

## Abstract

**Background:**

As the Coronavirus disease 2019 (COVID-19) pandemic continues to evolve, physicians must be equipped with adequate knowledge, skills on the prevention measures, and confidence in diagnosing and treating COVID-19 patients. Therefore, it is of great interest to assess the knowledge and practices of Physicians to identify existing gaps and improve occupational safety and viral surveillance.

**Methods:**

A cross-sectional study was conducted in Lebanon between 28th March and 11th April 2020. Data was collected through an online survey that included information on socio-demographic characteristics, knowledge, practice, physicians fear towards COVID-19 as well as their perceptions regarding actions/policies implemented by the Ministry of Public Health (MOPH) and their health care facilities. Multivariable logistic regression analyses were carried out to identify the factors associated with good knowledge of COVID-19 and good practice toward its prevention. Adjusted odds ratio and their 95% confidence intervals were reported.

**Results:**

Our survey revealed that the majority of Lebanese physicians had good knowledge about the disease (89.5%) while approximately half of the respondents adopted good preventive practices (49.7%). The odds of having good knowledge was 2.16 times higher among physicians aged 40 and above (adjusted OR = 2.16 with a 95% confidence interval (CI) of 1.08 to 4.34) compared to their counterparts aged less than 40 years old. Our results also showed that the odds of good practice was 2 times higher among frontline compared to the second line workers (adjusted OR = 2.01 with 95% CI of 1.21 to 3.34). Physicians with an experience of 10 years and above were 3.35 times more likely to have good practice compared to their counterparts (adjusted OR = 3.35 with 95% CI of 1.60 to 7.02). Finally, participants with good knowledge of COVID-19 were 2.04 times more likely to have a good practice (OR = 2.04 with 95% CI of 1.01 to 4.12).

**Conclusion:**

Lebanese physicians revealed a good level of knowledge; however, they had limited comprehension of the precautionary measures that protect them from this virus. Our findings have important implications for the development of strategies suitable for improving the level of practice among physicians and enhance prevention programs.

## Background

Novel coronavirus disease 2019 (COVID-19), which first emerged in China in December 2019, has turned into a worldwide disaster affecting at a rapid pace all the countries over the world [1, 2]. The latest figures, at the time of writing, show more than 17.5 million cases worldwide with a death toll exceeding 680.000 [3]. Given the nature and burden of COVID-19 pandemic, unprecedented challenges have faced governments, communities, and health care systems.

Physicians, who are directly engaged in the diagnosis, treatment, and care of COVID-19 patients, are exposed to infection from aerosol and droplet contamination [4] and at high risk for nosocomial infections [5]. As COVID-19 pandemic continues, the death of physicians has been increasingly reported worldwide. A recent cross-sectional study published in April 2020 showed that 120 medical doctors have died due to COVID-19; 67 in Italy, 34 in China, 6 in France, 4 in the United Kingdome (UK), the United States of America (USA), and Spain and 1 in South Korea [6].

Lebanon, a small Arab country, is taking part in the global fight against the COVID-19 pandemic. The first case was detected on February 21, 2020, in a traveler who had returned from Iran. As per August 1st, 2020, the Ministry of Public Health (MOPH) announced that the number of cases has reached 4730 [7]. During this outbreak, 207 health care workers (HCWs) of which 47 physicians had contracted the infection in their health care facilities. Most of the cases occurred in the early period of the outbreak due to misdiagnosis of the cases and the inadequate preventive practices. Thus, a preparedness of frontline physicians should be the main priority of all Lebanese health care settings to function properly and competently combat COVID-19. They should be equipped with adequate knowledge, skills on the prevention measures, and confidence in diagnosing and treating COVID-19 patients.

As part of an epidemic preparedness plan, it is of great interest to assess the knowledge and practices of physicians to identify existing gaps and improve occupational safety and viral surveillance. Thus, this study was conducted in the early stage of the COVID-19 outbreak in Lebanon to assess the knowledge and practices of Physicians regarding COVID-19. Also, we sought to evaluate their fear towards COVID-19 and their perceptions regarding policies/actions implemented by the MOPH and their health care settings in handling COVID-19 pandemic.

## Methods

### Study design and population

A cross-sectional study, using an online survey, was conducted during the early phase of the COVID-19 epidemic in Lebanon between 28th March and 11th April 2020. As the Lebanese government recommended the public to minimize face-to-face interaction and isolate themselves at home, potential respondents were electronically invited to participate. Thus, an online questionnaire using a Google form was distributed through “WhatsApp” groups and social media using a snowball technique. All physicians, working in hospitals or medical centers in different regions in Lebanon and who agreed to participate in the study, were included. No exclusion criteria were applied. Participants were identified via professional groups and academic institutions.

### Sample size calculation

The sample size was calculated using the online RAOSOFT sample size calculator designed specifically for population surveys. Based on an estimated population of 10,918 physicians [8], an anticipated response of 50%, a confidence level of 95% and a 5% margin of error, the required sample size would be at least 372.

### Instrumentation

A structured questionnaire was initially developed and designed by the authors in the English language to cover important aspects of knowledge and practice towards COVID-19 among physicians (Additional file 1). Core dimensions and items content of these two domains were identified through a review of the published literature on Middle East respiratory syndrome coronavirus (MERS-COV) [9–12] in addition to the most recent available information on COVID-19 from the World Health Organization (WHO) and the Centers for Disease Control and Prevention (CDC) websites up to 25th March 2020. Content validity of the resulting version was assessed by a panel of three experts with expertise in implementing infection control procedures and emergency preparedness. They were asked to evaluate the relevance of the items in assessing the knowledge and practices of physicians towards COVID-19. A consensus was reached after omitting four items that were rated irrelevant also minor linguistic edits were made. Then, the items were translated and adapted to the Arabic language by three translators. A final questionnaire was generated and was divided into five sections:

1. Socio-demographic information including age, gender, marital status, specialty, place of work, and clinical experience. Participants were also asked whether they were directly engaged in providing care to suspected, probable, or confirmed COVID-19 cases. Those who responded “yes” were considered as frontline physicians. Physicians, who answered “No” were considered as second-line workers.
2. Knowledge section: Six dimensions with a total of 19 items were designed to measure physician’s knowledge about nature of the disease (4 items), the transmission of the disease (3 items), actions in dealing with suspected, probable and confirmed cases (5 items), precautionary measures by health care providers (2 items), and treatment of the disease (5 items). All the items were answered on a true/false basis and an additional “do not know” option. A correct answer was assigned 1 point and an incorrect/unknown answer was assigned 0 points. The total knowledge score, obtained by the sum of the scores, ranged between 0 and 19. Based on Bloom’s cut off point, physicians’ overall knowledge was categorized as good if the score was above 60% (≥12 points) and poor if the score was less than 60% (<12 points) [13]. A question exploring the source of their knowledge concerning COVID19 was also added to this section. On this question, multiple responses from the participants were allowed.
3. Practice sections: seven questions were used to evaluate the uptake of various preventive measures. The items were answered “always”, “occasional” and “never” respectively. The answer (always) was assigned 1 point while answers (occasional and never) were assigned 0 points. The overall practice score, obtained by the sum of the scores, ranged between 0 and 7. Practice levels were defined as “good” or “poor” based on Bloom’s cut off point. Physicians with scores ≥80% (≥6 points) were classified as having a good practice, while those with scores < 80% (<6 points) were considered having poor practice [14].
4. Physicians’ fear towards COVID-19 was assessed by 2 items (I am afraid of working in places where patients suspected of COVID-19 are admitted/cared for, I am afraid of treating a patient with COVID-19) on a 3-point Likert scale (1- agree, 2- neutral, 3-disagree). A point of 1 was given to agree to answer while disagree or neutral responses were given a 0 point.
5. Physicians’ perceptions regarding policies/actions implemented by the Ministry of Public Health (MOPH) and their health care settings in handling the COVID-19 epidemic (1- appears in disarray/disorganized, 2- Insufficient, 3- acceptable/appropriate, 4- excessive and unnecessary.

### Pilot study

The survey was pilot tested in a sample of 5 physicians to check the clarity and readability of all items. Physicians did not report any problems in understanding the questionnaire. On average, the survey was completed within approximately 7 min. The data of the pilot study was removed from the final analysis.

### Data collection

Data was collected using an online survey. An invitation letter, including a link to the web survey platform, requesting Lebanese physicians to participate in this survey was prepared. The invitation letter includes information describing the survey and asking for voluntary participation of physicians as well as declarations of confidentiality and anonymity, and instructions for filling in the questionnaire. This letter was sent to approximately 1000 physicians through professional groups and academic institutions.

### Statistical analysis

Statistical analysis was carried out using the statistical software SPSS (Statistical Package for Social Sciences), version 22.0. Descriptive statistics were reported using means and standard deviations (SD) for continuous variables and frequency with percentages for categorical variables. Both bivariate and multivariable logistic regression analyses were performed to identify associated factors of good knowledge and good practice. The variables in bivariate analysis with *p*-value < 0.2 were entered into multivariable logistic regression. Adjusted odds ratio and their 95% confidence intervals were reported. The final logistic regression model was reached after ensuring the adequacy of our data using the Hosmer and Lemeshow test. The statistical significance level was set at p-value < 0.05 (two-sided).

## Results

### Baseline characteristics of the study participants

A total of 380 physicians participated in the survey among them 63.2% were males. The mean age was 40.4 (SD = 12.0) ranging from 24 to 80 years. The majority of the participants were specialists (85.5%) and 77.4% of respondents were frontline workers who were directly engaged in taking care of suspected, probable, or confirmed cases of COVID19. Almost half of the physicians (48.2%) had been practicing medicine for 10 years or longer. The summary of characteristics is shown in Table 1.

**Table 1:** Baseline characteristics of the study participants

	n (%)
**Gender**	
Male	240 (63.2)
Female	140 (36.8)
**Age categories (years)**	
<40	192 (50.5)
≥ 40	188 (49.5)
**Marital status**	
Married	263 (69.2)
Single	104 (27.4)
Widowed/divorced	13 (3.5)
**Specialty**	
Internal medicine	88 (23.2)
Surgery	79 (20.8)
Emergency medicine	65 (17.1)
General practitioner	55 (14.5)
Pediatrics	32 (8.4)
Obstetrics Gynecology	18 (4.7)
Others	43 (11.3)
**Place of work**	
Private hospitals	314 (82.6)
Public hospitals	66 (17.4)
**Frontline worker**	
No	86 (22.6)
Yes	294 (77.4)
**Years of experience**	
<10 years	197 (51.8)
≥ 10 years	183 (48.2)

n frequency, % percentage

### Assessment of Physician’s knowledge towards coronavirus

Out of the 380 physicians, the majority of 340 (89.5%) had good knowledge. Table 2 describes physicians’ answers towards COVID-19 knowledge items. Most of the respondents were aware of the nature of the disease (93.5%). Knowledge was also good for responses about the treatment of the disease (75.6%). Poor knowledge was more apparent in response to questions regarding the transmission of the disease (68.5%), actions in dealing with suspected, probable and confirmed cases (62.7%), and precautionary measures by health care providers (57.8%). The mean total knowledge score was 13.9 (SD = 1.8). There was no statistically significant difference between specialists and GPs as well as between frontline and second-line workers concerning knowledge (Data not shown). The main source of information was world health organization (WHO) (84.7%), followed by Ministry of Public Health (MOPH) (70.5%), CDC (41.0%), infectious diseases society of America (IDSA) (31%), television (18.4%) and Facebook (16.3%).

**Table 2:** Physicians’ responses to knowledge items (*N* = 380)

Knowledge items	Physicians‘answers		
	Correct	Wrong	Do not know
**Dimension 1: Nature of the disease (93.5%)**			
K1. The incubation period of Corona is 2–14 days (Yes)	374 (98.4)	4 (1.1)	2 (0.5)
K2. Recommended diagnostic approach in human is sampling of upper and lower airways secretions and PCR (polymerase chain reaction) examination (Yes)	374 (98.4)	5 (1.3)	1 (0.3)
K3. Covid-19 can be eliminated with at least 60% alcohol (Yes)	326 (85.8)	53 (13.9)	1 (0.3)
K4. The coronavirus can survive for many hours or many days in the environment (Yes)	348 (91.6)	25 (6.6)	7 (1.8)
**Dimension 2 Transmission of Disease (68.5%)**			
k.5 Covid-19 is transmitted through direct contact with respiratory tract secretions (Yes)	349 (91.8)	30 (7.9)	1 (0.3)
k.6 Covid-19 can be transmitted by transfusion of infectious blood and by needle stick injuries (No)	266 (70.0)	65 (17.1)	49 (12.9)
K7. Covid-19 can be transmitted through eating undercooked meat/chicken (No)	166 (43.7)	179 (47.1)	35 (9.2)
**Dimension3 Actions in dealing with suspected, probable and confirmed cases (62.7%)**			
K8. The use of personal protective equipment is necessary during aerosol production procedures, such as suction sputum sampling and intubation (Yes)	368 (96.8)	10 (2.6)	2 (0.5)
K9. Suspected cases of Covid-19 infection after triage should be taken into care in a negative pressure respiratory isolation room (No)	60 (15.8)	307 (80.8)	13 (3.4)
K10. The use of N95 masks is necessary when sampling of induced sputum from patients suspected of Covid-19 infection (Yes)	364 (95.8)	10 (2.6)	6 (1.6)
K11. Patients with Covid-19 infection admitted to an isolation room should use a surgical mask when moving and leaving the room for diagnostic and therapeutic procedures (Yes)	298 (78.4)	71 (18.7)	11 (2.9)
K12. All surfaces contaminated by the patients with Covid-19 infection should be cleaned with diluted (5%) bleaching solution (No)	102 (26.8)	195 (51.3)	83 (21.8)
**Dimension 4: Precautionary measures by health care Providers (57.8%)**			
K13. Droplet precautions should be followed by health care providers in dealing with suspected, probable and confirmed cases of Covid-19 infection (Yes)	330 (86.8)	16 (4.2)	34 (8.9)
K14. Airborne precautions should be followed by health care providers in dealing with suspected, probable and confirmed cases of Covid-19 infection (No)	110 (28.9)	262 (68.9)	8 (2.1)
**Dimension 5: Treatment of the disease (75.6%)**			
K15. Oxygen therapy should be given to all cases of severe Covid-19 with acute respiratory infection (Yes)	277 (72.9)	84 (22.1)	19 (5.0)
K16. Antibiotic therapy is required for the treatment of pneumonia until confirmation of suspected cases of Covid-19 infection (Yes)	157 (41.3)	191 (50.3)	32 (8.4)
K17. Ventilation with an endotracheal tube must be carried out in patients with confirmed or suspected coronaviruses with clinical manifestations of acute respiratory distress syndrome (Yes)	316 (83.2)	43 (11.3)	21 (5.5)
K18. High doses of systemic corticosteroids should be avoided in patients with confirmed or suspected Covid-19 infection and clinical manifestations of viral pneumonia (Yes)	321 (84.5)	27 (7.1)	32 (8.4)
K19. There is no currently effective cure for Covid-19, but early symptomatic and supportive treatment can help most patients recover from the infection (Yes)	366 (96.3)	8 (2.1)	6 (1.6)

Yes /No in the parentheses denotes the intended (correct) answer

### Assessment of Physician’s practice towards coronavirus

Table 3 shows the percentage of good practices regarding the various preventive measures. The average score of practice was 5.4 (SD = 1.1, ranged 1–7). Cleaning my hands with hydro-alcoholic gel during your work shift was the most prevalent behavior (97.9%), followed by washing hands during work shift (96.6%), wearing a mask (89.5%) and gloves (65.3%). Only half of our participants can maintain physical distancing of at least 1.5 m from colleagues (55.3%). In addition, only 75.5 and 61.8% of the physicians were aware of the order of putting on and removing personal protective equipment (PPE). Of all physicians, nearly half (49.7%) reported taking good preventives practices while performing their job.

**Table 3:** Physicians’ correct responses regarding preventive practices towards COVID-19 (*N* = 380)

	N	%
P1- I wear a mask while performing my job	340	89.5
P2- I wear gloves while performing my job	248	65.3
P3- I wash my hands during your work shift	367	96.6
P4- I rub my hands with hydro-alcoholic gel during your work shift	372	97.9
P5- I can maintain physical distancing of at least 1.5 m from colleagues?	210	55.3
P-6. I put on properly (Don) my PPE^a^: 1- gown, 2- mask, 3- gloves.	287	75.5
P-7. I remove properly (Doffing) my PPE^a^: 1- gloves, 2- do hand hygiene, 3- gown, 4- mask	235	61.8

N Frequency, % percentage, ^a^ Personal protective equipment

### Assessment of physicians’ fears towards COVID-19

Table 4 describes physicians’ answers towards parameters related to fear of COVID-19. Of all physicians, 32.6% exhibited fears towards working in places where patients suspected of COVID-19 infection are admitted and 36.3% reported that they were afraid of treating a patient with COVID-19 infection.

**Table 4:** Physicians’ responses to fear items (*N* = 380)

	Afraid	Neutral	Unafraid
I am afraid of working in places where patients suspected of COVID-19 infection are admitted/cared for.	124 (32.6)	10 (2.6)	246 (64.7)
I am afraid of treating a patient with COVID-19 infection.	138 (36.3)	12 (3.2)	230 (60.5)

Results are expressed in terms of frequency and percentage

### Physicians’ perceptions towards implemented policies/actions in fighting COVID-19

**Fig. 1:**
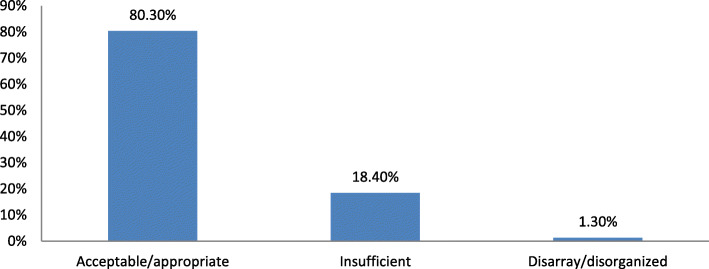
Physicians’ Perceptions towards policies/actions implemented by the ministry of Public health in fighting COVID-19

**Fig. 2:**
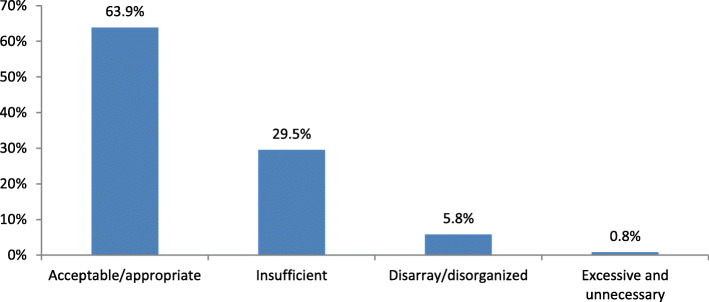
Physicians’ Perceptions towards policies/actions implemented by health care facilities in fighting COVID-19

The majority of participants (80.3%) declared that policies/actions implemented by the ministry of public health are adequate (Fig. 1), whereas only 63.9% revealed that the policies/actions implemented by their health care facilities were adequate in fighting COVID-19 (Fig. 2).

### Factors associated with good knowledge on COVID-19

Results of the bivariate and multivariable analysis showed that only age was significantly associated with good knowledge at *p*-value <0.05. The odds of having good knowledge was 2.16 times higher among physicians aged 40 and above (adjusted OR = 2.16 with a 95% CI of 1.08 to 4.34) compared to their counterparts aged less than 40 years old (Table 5).

**Table 5:** Factors associated with good knowledge toward COVID-19 (*N* = 380)

	Knowledge			
	Poor	Good	*p*-value	Adjusted OR (95%CI)
**Gender**			0.55	
Male	27 (67.5)	213 (62.3)		
Female	13 (32.5)	127 (37.4)		
**Age categories (years)**			0.023*	
<40	27 (67.5)	165 (48.5)		**1.00**
≥ 40	13 (32.5)	175 (51.5)		2.16 (1.08–4.34)*
**Marital status**			0.80	
Married	27 (67.5)	236 (69.4)		
Others	13 (32.5)	104 (30.6)		
**Specialty**			0.3	
General	8 (20.0)	47 (13.8)		
Practitioner	32 (80.0)	293 (86.2)		
**Place of work**			0.18	
Private hospitals	30 (75.0)	284 (83.5)		1.00
Public hospitals	10 (25.0)	56 (16.5)		0.62 (0.28–1.34)
**Frontline worker**			0.44	
No	11 (27.5)	75 (22.1)		
Yes	29 (72.5)	265 (77.9)		
**Years of experience (years)**			0.45	
<10	23 (57.5)	174 (51.2)		
≥ 10	17 (42.5)	166 (48.8)		

*p-value<0.05 is considered significant

### Factors associated with good practice toward COVID-19 prevention

Table 6 represents the Multivariable logistic regression. Our results showed that the odds of good practice was 2 times higher among frontline compared to the second line workers (adjusted OR = 2.01 with 95% CI of 1.21 to 3.34). Physicians with an experience of 10 years and above were 3.35 times more likely to have good practice compared to their counterparts (adjusted OR = 3.35 with 95% CI of 1.60–7.02). Finally, participants with good knowledge toward COVID-19 were 2.04 times more likely to have a good practice (adjusted OR = 2.04 with 95% CI of 1.01 to 4.12).

**Table 6:** Factors associated with good practice toward COVID-19 prevention (*N* = 380)

	Practice			
	Poor	Good	*p*-value	Adjusted OR (95%CI)
**Gender**			0.72	
Male	119 (62.3)	121 (64.0)		
Female	72 (37.7)	68 (36.0)		
**Age categories (years)**			0.08	
<40	105 (55%)	87 (46%)		1.00
≥ 40	86 (45.0)	102 (54.0)		0.51 (0.24–1.07)
**Marital status**			0.11	
Married	125 (65.4)	138 (73.0)		
Others†	66 (34.6)	51 (27.0)		1.00
**Specialty**			0.32	0.86 (0.52–1.40)
General	31 (16.2)	24 (12.7)		
Practitioner	160 (83.8)	165 (87.3)		
**Place of work**			0.75	
Private hospitals	159 (83.2)	155 (82.0)		
Public hospitals	32 (16.8)	34 (18.0)		
**Frontline worker**			0.008*	
No	54 (28.3)	32 (16.9)		**1.00**
Yes	137 (71.7)	157 (83.1)		**2.01 (1.21–3.34)**
**Years of experience (years)**			0.001*	
<10	115 (60.2)	82 (43.4)		**1.00**
≥ 10	76 (39.8)	107 (56.6)		**3.35 (1.60–7.02)**
**Knowledge**			0.049*	
Poor	26 (13.6)	14 (7.4)		**1.00**
Good	165 (86.4)	175 (92.6)		**2.04 (1.01–4.12)**

† others included single, widowed, and divorced, *p-value<0.05 is considered significant

## Discussion

Since the declaration of the first case on February 21st,2020, a great public health concern emerged in Lebanese public and governmental institutions. Until this date, no definitive treatment was recommended, and physicians are expected to play an important role in the detection and management of cases of COVID-19. In addition, they are carrying the burden to prevent further spreading of the disease. Thus, lack of their knowledge regarding transmission and clinical manifestations of the disease as well as inadequate preventive practices could lead to misdiagnosis of the case and increase the risk of infection.

This study was conducted during the early stage of the COVID-19 outbreak in Lebanon to provide insight into the knowledge and practices of physicians. Results of our survey revealed that the majority of Lebanese physicians had good knowledge about the disease while only half of the respondents adopted good preventive practices. Our results also showed that frontline physicians who had been practicing medicine for more than 10 years, and with a good level of knowledge had good practice compared to their counterparts.

Our finding of a good level of knowledge among physicians is in line with that of Minghe Zhou et al., who reported that 89.7% of HCWs have sufficient knowledge regarding COVID-19 with doctors showing higher scores compared to nurses and paramedics [15]. When looking at the dimensions of knowledge, we found that most participants were aware of the nature of the disease (93.5%) and its treatment (75.6%). However, a poor level of knowledge was clearly shown in response to the questions regarding the transmission of the disease (31.5%), similarly for the actions when dealing with COVID-19 cases (37.3%) and precautionary measures by health care providers (42.2%). Consistent with our findings, Akshaya Srikanth Bhagavathula et al., [16] reported a poor level of knowledge among HCWs concerning the transmission of the disease (39%). This could be attributed to the scientific dilemma proposed by the experts regarding this topic. Logistic regression analysis showed that the age of the participants was the only significant predictor of good knowledge. This comes inconsistency with the study conducted in Pakistan to evaluate knowledge, attitude, practice, and perceived barriers among HCWs regarding COVID-19 by Saqlain et al. [17].

We also found that physicians used official international and governmental websites such as WHO (85.0%), MOPH (70.5%), CDC (41.0%), and (IDSA) (31%) as main sources of information about COVID-19. This indicates that physicians utilize reliable sources to acquire information regarding COVID-19 and reflect their good level of knowledge. It is also worth mentioning that some physicians used TV (18.4%) and Facebook (16.3%) as sources of information. Although these platforms provide an easy way to get the information, they can also be a source of fake news. Thus, it is highly recommended for physicians to seek information from scientific and authentic platforms.

Concerning practice, approximately half of the respondents (49.7%) followed infection control practices. These include regular hand hygiene (97%), wearing a face mask (89%), and gloves (65%). Only half of our participants can maintain a social distancing of at least 1.5 m from colleagues (55%). This could be to overcrowding or small surfaces in health care settings. In addition, only 77.5 and 61.8% of the physicians were aware of the proper donning and doffing PPE. A recent study conducted in Pakistan showed that 91.4% of physicians had good practices in following precautions to avoid COVID-19 [18].

Limited resources in the institution, the lack of experience, the poor level of knowledge regarding mode of transmission of the disease, actions are taken when dealing with cases and precautionary measures could partly explain the poor preventive practices of physicians. The Lebanese order of physicians (LOP), the syndicate of hospitals, and the scientific societies have conducted many online training sessions for HCWs mainly physicians. In addition, several protocols and memo regarding SARS-Cov-2 were issued. Despite all of this, a significant number of HCWs have been infected due to misdiagnosis of the cases and inadequate preventive practices. Thus, continued professional education and training are advised to empower physicians by supporting their ability to acquire and use evidence-based information. This imposes an action plan from LOP and syndicate of hospitals to enhance the actions and preventive measures that should be implemented when confronting a novel virus.

Similar to the findings of Zhang et al., [15], results of our survey showed that frontline physicians who had been practicing medicine for more than 10 years had better practice compared to their counterparts. This indicates that frontline physicians’ with more than 10 years of experience had skills to deal with public health emergencies and are confident in their ability to defeat the virus. A finding of considerable concern in this survey is that more than 30% of the respondents expressed their fear towards treating a patient with COVID-19 which in turn was associated with poor practice. Indeed, SARS-COV-2 is highly contagious which could explain the reluctance of physicians to treat patients with COVID-19. Thus, psychological interventions to improve physicians’ mental health and to enhance confidence in their ability to treat patients are needed. With a deeper understanding of COVID-19, we believe that physicians’ fear will decrease and the number of physicians who are willing to treat these patients would gradually increase.

Interestingly, the majority of participants (80.3%) declared that the policies/actions implemented by the MOPH are adequate. The Lebanese governments have set early lockdown measures such as the closure of all educational institutions, international airport, and its sea borders in addition to the nighttime curfew. All these measures have contributed, till the time of the writing, to the success in slowing the pace of COVID-19 progression. However, only 63.9% revealed that the policies/actions implemented by their health care facilities were adequate in fighting COVID-19. This could be due to the poor infection control practices implemented in the health care facilities and the shortage of available PPE for all HCWs. Thus, increasing the preparedness of all health care facilities is vital to increase the confidence of physicians so to improve their work.

The findings of the present study should be considered in light of several limitations. Firstly, no validated tool for the assessment of the knowledge and practices of HCWs was available. We have adapted and modified tools used for the assessment of knowledge, attitude, and practice toward MERS-COV [9–12] in addition some items were formulated from WHO and CDC guidelines. Secondly, due to the lockdown, we did not design the sample to statistically represent the Lebanese population of physicians and make rigid extrapolations, but to offer for the first time, useful insights of the knowledge and practices towards COVID-19. Thirdly, only physicians who publicly shared their phone numbers were eligible to participate; this could have led to selection bias. Therefore, assessment of knowledge and practices of a significant proportion of physicians and their opinions might be missed in this analysis. Fourth, some participants might have provided socially desirable responses rather than their actual opinions.

## Conclusion

This study offers useful insights into the knowledge and practices of Lebanese physicians towards COVID-19. Lebanese physicians revealed a good level of knowledge; however, they exhibit poor preventive practices. As the global threat of COVID-19 continues to emerge, there is a clear need for further education and training, particularly on disease transmission, actions in dealing with COVID-19 cases, and preventive measures. This should, in turn, improve their confidence and relief their fears towards getting infected by COVID-19 cases.

## Supplementary information

**Additional file 1.** Study questionnaire.

## Data Availability

Data are available from the corresponding authors upon reasonable request.

## Abbreviations

SARS-Cov-2: Severe acute respiratory syndrome coronavirus 2
COVID-19: Coronavirus disease 2019
HCWs: Health care workers
NHCPRC: National Health Commission of the People’s Republic of China
CDC: Centers of disease controlled and prevention
MOPH: Ministry of Public Health
IRB: Institutional Review board
ZHUMC: Al Zahraa Hospital University Medical Center
MERS-COV: Middle East respiratory syndrome coronavirus
WHO: World Health Organization
SPSS: Statistical Package for Social Sciences
SD: Standard deviations
CI: Confidence interval
IDSA: Infectious diseases society of America
TV: Television
LOP: Lebanese order of physicians
PPE: Personal protective equipment
