# Supplementary material for: Knowledge and practice of physicians during COVID-19 pandemic: a cross-sectional study in Lebanon
Source: BMC Public Health. 2020 Sep 29;20:1474. doi: 10.1186/s12889-020-09585-6 (PMC7523262; doi:10.1186/s12889-020-09585-6)
Supplement: Supplementary file 1 — Additional file 1. Study questionnaire. [file 12889_2020_9585_MOESM1_ESM.docx]

**Questionnaire**

1. **Baseline characteristics of the study participants**

|  |
| --- |
| **Gender** |
| Male |
| Female |
| **Age categories (years)** |
| ˂40 |
| ≥40 |
| **Marital status** |
| Married |
| Single |
| Widowed/divorced |
| **Specialty** |
| Internal medicine |
| Surgery |
| Emergency medicine |
| General practitioner |
| Pediatrics |
| Obstetrics Gynecology |
| Others |
| **Place of work** |
| Private hospitals |
| Public hospitals |
| **Frontline worker** |
| No |
| Yes |
| **Years of experience** |
| ˂10 years |
| ≥10 years |

1. **Physicians’ knowledge towards COVID-19**

| **Knowledge items** | **Physicians‘answers** | | |
| --- | --- | --- | --- |
|  | **Yes** | **No** | **Do not know** |
| **Dimension 1: Nature of the disease** | | | |
| K1. The incubation period of Corona is 2–14 days |  |  |  |
| K2. Recommended diagnostic approach in human is sampling of upper and lower airways secretions and PCR (polymerase chain reaction) examination |  |  |  |
| K3. Covid-19 can be eliminated with at least 60% alcohol |  |  |  |
| K4. The coronavirus can survive for many hours or many days in the environment |  |  |  |
| **Dimension 2 Transmission of Disease** | | | |
| k.5 Covid-19 is transmitted through direct contact with respiratory tract secretions |  |  |  |
| k.6 Covid-19 can be transmitted by transfusion of infectious blood and by needle stick injuries |  |  |  |
| K7. Covid-19 can be transmitted through eating undercooked meat/chicken |  |  |  |
| **Dimension3 Actions in dealing with suspected, probable and confirmed cases** | | | |
| K8. The use of personal protective equipment is necessary during aerosol production procedures, such as suction sputum sampling and intubation |  |  |  |
| K9. Suspected cases of Covid-19 infection after triage should be taken into care in a negative pressure respiratory isolation room |  |  |  |
| K10. The use of N95 masks is necessary when sampling of induced sputum from patients suspected of Covid-19 infection |  |  |  |
| K11. Patients with Covid-19 infection admitted to an isolation room should use a surgical mask when moving and leaving the room for diagnostic and therapeutic procedures |  |  |  |
| K12. All surfaces contaminated by the patients with Covid-19 infection should be cleaned with diluted (5%) bleaching solution |  |  |  |
| **Dimension 4: Precautionary measures by health care Providers** | | | |
| K13. Droplet precautions should be followed by health care providers in dealing with suspected, probable and confirmed cases of Covid-19 infection |  |  |  |
| K14. Airborne precautions should be followed by health care providers in dealing with suspected, probable and confirmed cases of Covid-19 infection |  |  |  |
| **Dimension 5: Treatment of the disease** | | | |
| K15. Oxygen therapy should be given to all cases of severe Covid-19 with acute respiratory infection |  |  |  |
| K16. Antibiotic therapy is required for the treatment of pneumonia until confirmation of suspected cases of Covid-19 infection |  |  |  |
| K17. Ventilation with an endotracheal tube must be carried out in patients with confirmed or suspected coronaviruses with clinical manifestations of acute respiratory distress syndrome |  |  |  |
| K18. High doses of systemic corticosteroids should be avoided in patients with confirmed or suspected Covid-19 infection and clinical manifestations of viral pneumonia |  |  |  |
| K19. There is no currently effective cure for Covid-19, but early symptomatic and supportive treatment can help most patients recover from the infection |  |  |  |

1. **Physicians’ preventive practices towards COVID-19**

|  | **Never** | **Always** | **Occasional** |
| --- | --- | --- | --- |
| P1- I wear a mask while performing my job |  |  |  |
| P2- I wear gloves while performing my job |  |  |  |
| P3- I wash my hands during your work shift |  |  |  |
| P4- I rub my hands with hydro-alcoholic gel during your work shift |  |  |  |
| P5- I can maintain physical distancing of at least 1.5 meters from colleagues? |  |  |  |
| P-6. I put on properly (Don) my PPE^a^: 1- gown, 2- mask, 3- gloves. |  |  |  |
| P-7. I remove properly (Doffing) my PPE^a^: 1- gloves, 2- do hand hygiene, 3- gown, 4- mask |  |  |  |

1. **Physicians’ fear toward COVID-19**

|  | **Agree** | **Neutral** | **Disagree** |
| --- | --- | --- | --- |
| I am afraid of working in places where patients suspected of COVID-19 infection are admitted/cared for. |  |  |  |
| I am afraid of treating a patient with COVID-19 infection. |  |  |  |

1. **Physicians’ Perceptions towards policies/actions implemented by the ministry of Public health in fighting COVID-19**

- Acceptable/appropriate
- Insufficient
- Disarray/disorganized
- Excessive and unnecessary

1. **Physicians’ Perceptions towards policies/actions implemented by health care facilities in fighting COVID-19**

- Acceptable/appropriate
- Insufficient
- Disarray/disorganized
- Excessive and unnecessary
